# Case report: A rare case of breast and multiorgan metastases secondary to papillary thyroid carcinoma

**DOI:** 10.3389/fonc.2024.1422817

**Published:** 2024-11-04

**Authors:** Wenqin Huang, Yalong Yang, Peng Zhan, Liang Jiang, Jian Chen, Hongmei Zheng

**Affiliations:** ^1^ Department of Breast Surgery, Hubei Cancer Hospital, Tongji Medical College, Huazhong University of Science and Technology, National Key Clinical Specialty Construction Discipline, Hubei Provincial Clinical Research Center for Breast Cancer, Wuhan Clinical Research Center for Breast Cancer, Wuhan, Hubei, China; ^2^ Department of Head and Neck Surgery, Hubei Cancer Hospital, Tongji Medical College, Huazhong University of Science and Technology, Wuhan, Hubei, China

**Keywords:** Papillary thyroid carcinoma, breast secondary malignant tumor, *BRAF* gene, *RET* gene, case report

## Abstract

Papillary thyroid carcinoma (PTC) is generally considered a highly indolent endocrine malignancy, often accompanied by cervical lymph node metastasis and rarely involving distant metastases. We present a rare case of a 37-year-old woman with PTC, who exhibited regional lymph node metastasis, right breast metastasis, and probable right psoas major and multiple bone metastases. Initial symptoms included hoarseness, and subsequent examination revealed a secondary malignant tumor in the right breast, originating from the thyroid gland. This case highlights an unusual pattern of multiple systemic metastasis in PTC, particularly the rare occurrence of breast metastasis.

## Introduction

Monitoring and epidemiological data show that thyroid cancer is the most common endocrine malignant tumor, and the incidence in female patients is almost three times than that in male patients (1, 2). Among various histological types, papillary thyroid carcinoma (PTC) is the most common type, accounting for 70%–80% of all cases (3). PTC is usually known as an inert tumor, with a 10-year survival rate of approximately 93% (3, 4). The cancer-related deaths attributed to encapsulated non-invasive follicular PTC variants have not been reported, and the estimated risk of recurrence is less than 1% (1). Conversely, specific gene mutation-harboring PTC subtypes exhibit heightened aggressiveness, correlating with advanced tumor stages and lymph node metastases at initial diagnosis (1). Despite garnering heightened research interest and an expanding case repository for these aggressive variants, the occurrence of multiple metastases in PTC, particularly breast metastasis, remains an exceedingly unusual clinical phenomenon. Here, we present a case study of a 37-year-old female patient who developed breast and multiorgan metastases as a sequel to PTC.

## Case description

Informed consent was obtained from the patient, who signed a consent form.

**Figure 1:**
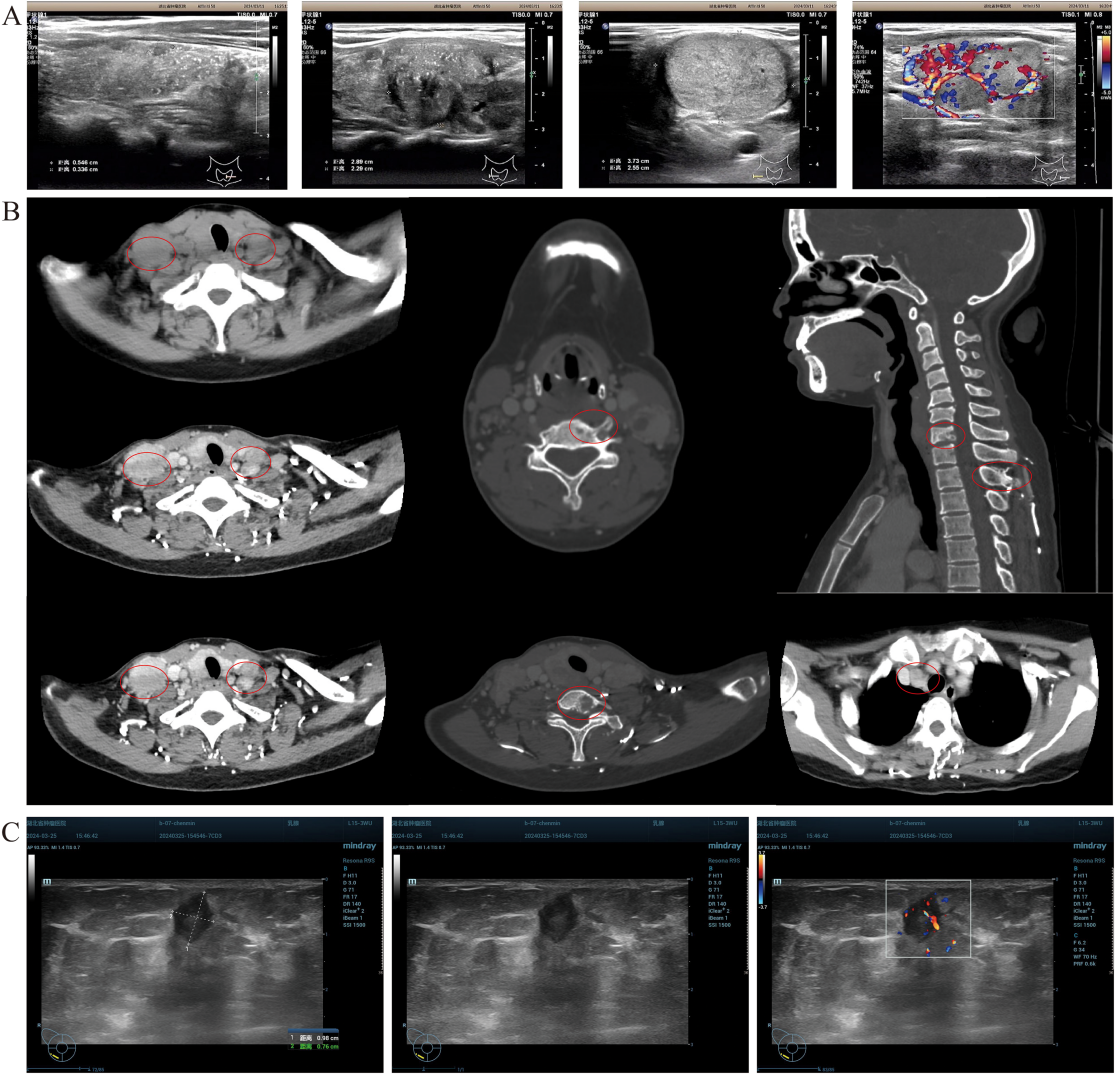
Patient imaging evaluation. **(A)** Thyroid color Doppler ultrasound revealed the presence of multiple punctate strong echoes within the bilateral lobes of the thyroid gland. These findings were accompanied by multiple hypoechoic and isoechoic masses, exhibiting indistinct boundaries and irregular contours. The internal echogenicity was heterogeneous, with evidence of multiple small calcifications. CDFI demonstrated discernible blood flow signals within these masses. Additionally, multiple lymph node echoes with well-defined boundaries but irregular shapes were identified in the bilateral cervical regions (zones II, III, IV, and VI). Some of these lymph nodes exhibited fusion, and the structure of the cortex and medulla was not clear. A subset of these lymph nodes displayed intense punctate echoes, and CDFI confirmed the presence of blood flow signals. **(B)** CT scan of the neck showed multiple lymph node metastasis in the anterior–superior mediastinum and multiple bone destruction in the lower cervical spine. **(C)** Breast color Doppler ultrasound examination of the right breast detected a 0.95 × 0.77 × 0.84 cm low-echoic mass, characterized by a clear boundary and an irregular shape. Certain sections of this mass exhibited angular features, with an aspect ratio > 1. CDFI analysis further revealed abundant blood flow signals within the mass.

**Figure 2:**
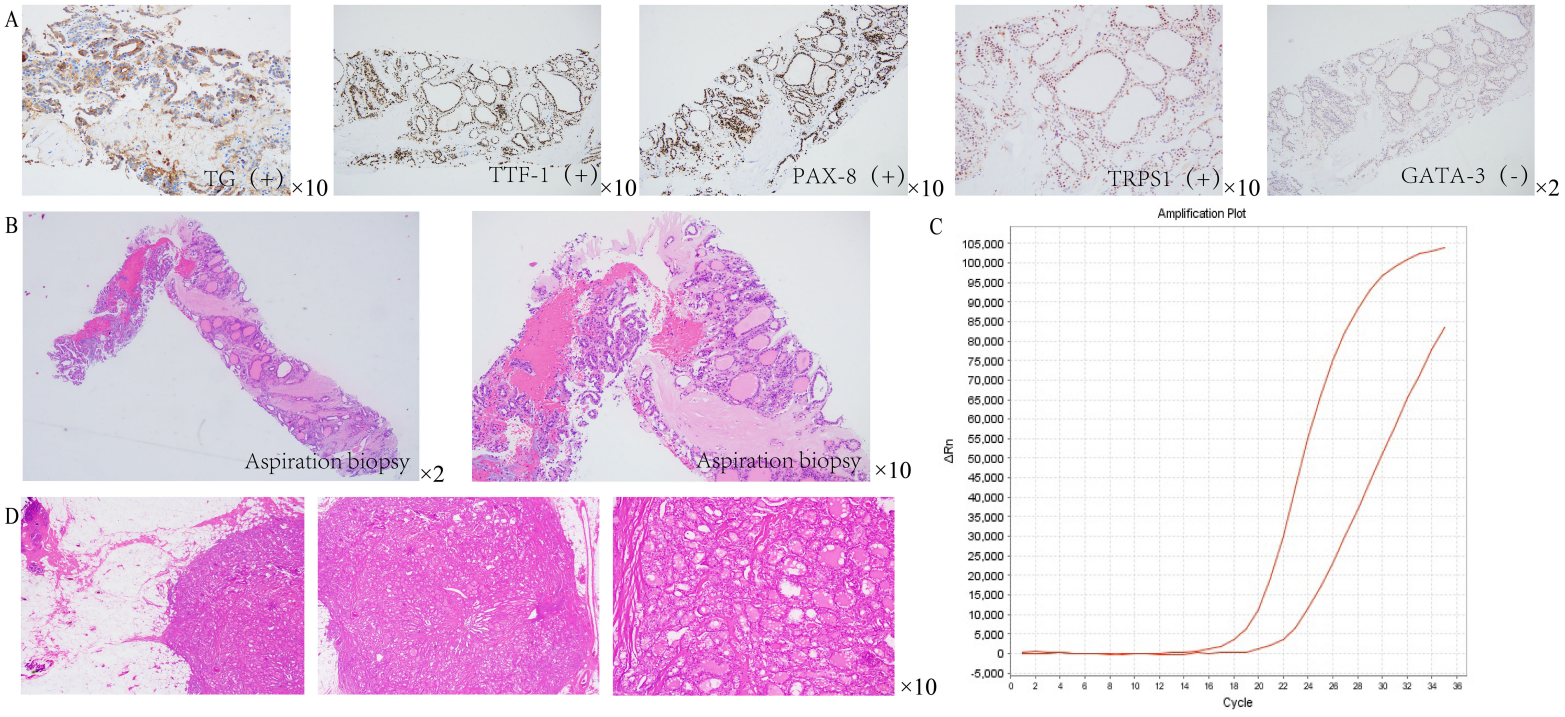
Pathological results of breast tissue in patients. **(A)** Immunohistochemical analysis revealed positive staining for thyroglobulin (Tg), thyroid transcription factor-1 (TTF-1), and PAX-8. Notably, TRPS1 staining was observed to be weakly positive, whereas GATA-3 staining was definitively negative. **(B)** HE staining showed that the right breast mass was consistent with adenocarcinoma. **(C)** PCR results indicated a positive fusion of the CCDC6-*RET* gene (Exon 1 to Exon 12). **(D)** HE staining of the resected specimen from the right breast mass confirmed the presence of (right breast) adenocarcinoma.

A 37-year-old female patient was diagnosed with PTC after initially presenting with hoarseness. In the preoperative routine examination, the physical examination revealed a hard, movable mass with a diameter of approximately 1.0 cm in contact with the left thyroid gland. This mass moved up and down with swallowing and was not tender. Upon physical examination, palpable multiple masses were observed within the right thyroid gland, with the largest measuring approximately 2.0 cm × 3.0 cm. Additionally, multiple enlarged lymph nodes were palpable on both sides of the neck, of which the larger one on the left side was approximately 3.0 cm in diameter, and the larger one on the right side was approximately 2.0 cm in diameter. Thyroid ultrasound revealed multiple punctate calcifications in both lobes, along with the presence of multiple nodules classified as TI-RADS 4c in both lobes and a follicular cyst in the left lobe categorized as TI-RADS 2. Furthermore, it indicated enlarged lymph nodes in bilateral II, III, IV, and VI zones of the neck, suggestive of metastatic lymph nodes (**Figure 1A**). Neck CT scanning demonstrated scattered nodules and masses bilaterally within the thyroid gland, prompting consideration of thyroid cancer. The scan also revealed extensive lymph node metastasis in bilateral Ib–V regions and the anterior–superior mediastinum, as well as multiple instances of bone destruction in the lower cervical spine, indicative of metastasis (**Figure 1B**). Breast color Doppler ultrasound examination identified a solid mass in the right breast, classified as BI-RADS 4b, prompting an ultrasound-guided biopsy for pathological assessment (**Figure 1C**). Histopathological analysis confirmed the presence of adenocarcinoma in the right breast mass, which, upon correlation with HE morphology and immunophenotyping, was consistent with a thyroid origin. Immunohistochemistry results showed TTF-1 (+), PAX-8 (+), TG (+), GATA-3 (−), TRPS1 (weak +), P53 (weak +, wild type expression), Ki67 (ClONE: SP6) (Li:2%), and *BRAF* (V600E) (−). CCDC6-*RET* (Exon 1–Exon 12) gene fusion was positive (**Figure 2**).

**Figure 3:**
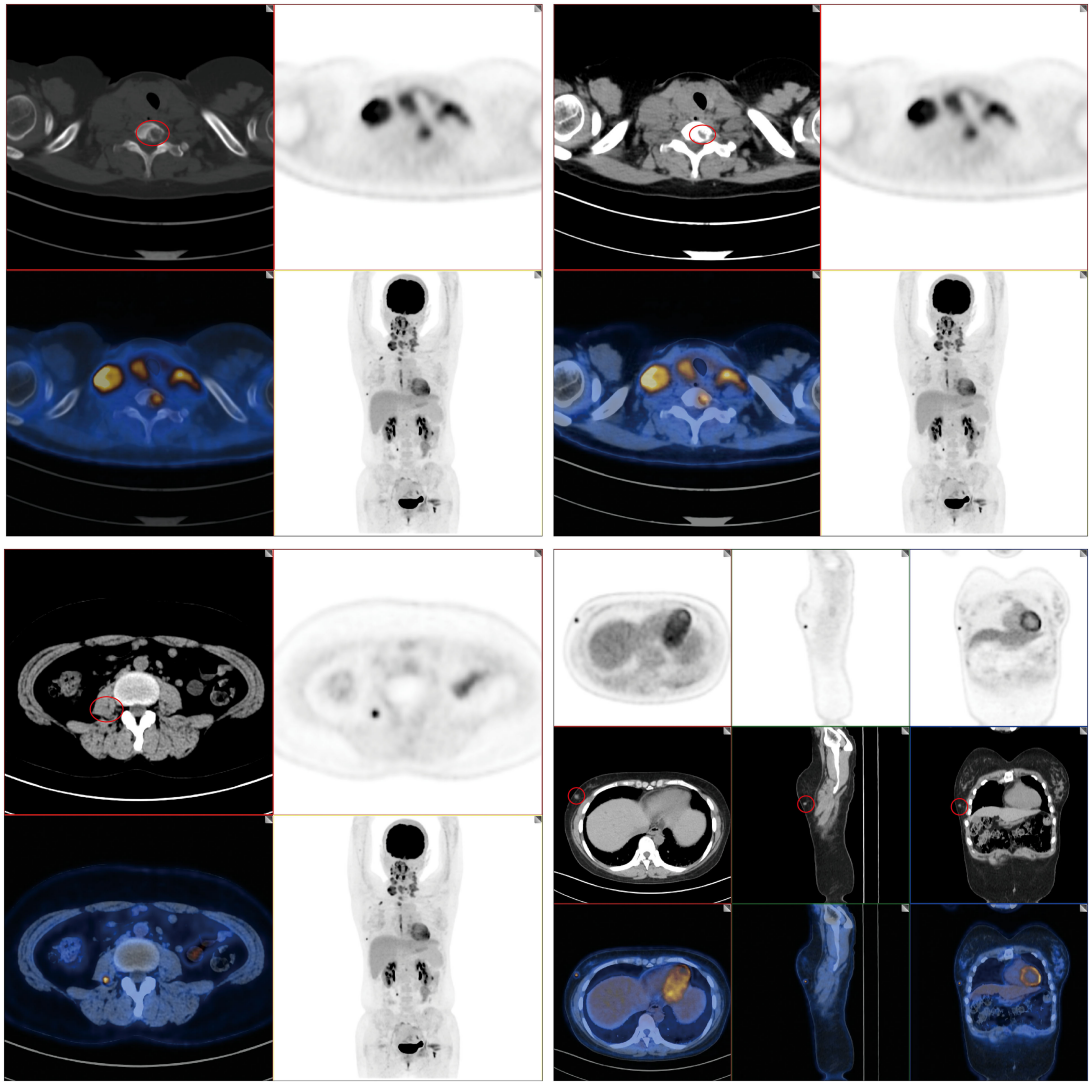
PET-CT results of patients. PET-CT imaging revealed multiple, slightly hypoechoic nodules within the bilateral thyroid lobes, suggesting the possibility of lymph node metastasis in the bilateral neck, supraclavicular region, and mediastinum. Furthermore, soft tissue density nodules were discernible in the outer lower quadrant of the right breast. Additionally, a nodular abnormal radioactive concentration shadow was observed in the right psoas major muscle; bone destruction was evident in the right second rib and seventh cervical vertebra.

Combined with the above suggested metastasis, in order to investigate whether there is metastasis in other distant organs of the patient, we advocate the utilization of positron emission tomography–computed tomography (PET-CT) imaging. The findings indicate the presence of multiple, slightly hypoechoic nodules within the bilateral lobes of the thyroid gland, prompting the consideration of thyroid cancer. Furthermore, the possibility of lymph node metastasis is entertained in the bilateral neck, supraclavicular region, and mediastinum, based on observed abnormalities. In the outer lower quadrant of the right breast, soft tissue density nodules were discernible, raising the suspicion of metastatic lesions, thereby not excluding breast cancer as a differential diagnosis. Additionally, an abnormal nodular radioactive concentration shadow in the right psoas major muscle suggests the likelihood of metastatic involvement. Bone destruction was also observed in the second rib and the seventh cervical vertebra on the right side, and bone metastasis was considered (**Figure 3**).

The patient underwent a comprehensive surgical procedure encompassing bilateral thyroidectomy coupled with dissection of bilateral central and cervical lymph nodes. Postoperative pathological examination revealed a papillary carcinoma of the common type, exhibiting intraglandular dissemination, with a little hobnail-like subtype (<1%). This subtype was further characterized by invasion into the thyroid capsule and extracapsular adipose tissue. Metastatic carcinoma was identified in 26 out of 57 lymph nodes examined. Immunohistochemical analysis indicated a negative *BRAF* (V600E) status in the thyroid cancer cells.

For breast metastatic lesions, without the excisional biopsy, the possibility of an independent tumor solely originating in the breast cannot be excluded. In such cases, we performed the local excision for biopsy, which can not only confirm its origin but also specify its nature (5). Therefore, the patient also underwent resection of the right breast mass under local anesthesia. Pathological examination suggested that (right breast) adenocarcinoma, combined with medical history and HE morphology, was consistent with the source of thyroid cancer. So far, the postoperative pathological examination results confirmed that the patient’s breast malignant tumor originated from a thyroid malignant tumor.

According to the current treatment principles for metastatic tumors (6), for breast metastatic lesions, only a local tumor resection is performed, without radiation therapy and chemotherapy related to metastatic breast cancer. After the tumor resection, it is clear that the metastatic breast cancer originates from thyroid cancer. According to the diagnosis and treatment suggestions for differentiated thyroid cancer (DTC), the patient is recommended to receive subsequent iodine-131 treatment.

Subsequently, the patient received iodine-131 treatment, and the condition was well controlled. Follow-up to August 2024, the patient lived in other provinces and did not receive any examination and treatment. The patient reported that the general condition was good, but the hoarseness was not improved.

## Discussion

Distant metastases in PTC are infrequent, manifesting in less than 10% of individuals diagnosed with DTC. Notably, half of these metastases are already present at the time of initial tumor detection, whereas the remaining instances may remain undetected until several decades after treatment (1). The incidence of thyroid cancer metastasis is heightened in patients harboring invasive histological subtypes, including the high cell, columnar, hobnail, and solid variants. The lung and bone are the most frequently implicated sites of metastasis, accounting for 49% and 25% of all cases, respectively. Concurrent involvement of both sites occurs in 15% of cases, while metastases to the brain, liver, and skin are comparatively rare (1). In clinical practice, metastatic dissemination to atypical organs, such as the breast, skin, eyes, pancreas, and skeletal muscle, is infrequently reported. The diagnosis of distant metastasis is typically predicated on the presentation of clinical symptoms or the detection of suspicious imaging findings. The overall mortality rates at 5 and 10 years subsequent to the diagnosis of distant metastasis are 65% and 75%, respectively (1).

In the case reported by Dris Kharroubi et al., there was a 19-year-old woman with multifocal typical PTC without local lymph node infiltration. After receiving surgical treatment and radioactive iodine treatment, breast metastases were found after a radioactive iodine scan (7). Compared with the case reported by Kharroubi et al., the local infiltration of the primary tumor in our case was more serious and there were more distant metastases. In the case reported by Dan Zhang et al., there was a middle-aged female patient with papillary thyroid oncocytoma with distant metastasis of the breast and cervical spine. In the case of multiple recurrences, she only received surgical treatment of the primary tumor and eventually died of cancer cachexia (8). The case we reported is similar to the case reported by Dan Zhang et al., which is also a middle-aged female patient with cervical lymph node infiltration and distant metastasis of breast and cervical spine. However, the primary tumor subtypes of the two are different, and the compliance is also different.

*BARF* and *RET* genes are associated with distant metastasis of PTC. *BRAF* V600E mutations are frequently observed in subsets of PTC, exhibiting more aggressive clinicopathological profiles, as reported in the literature (9). In the case of this patient, a positive CCDC6-*RET* (Exon 1–Exon 12) gene fusion was detected. *RET* kinase fusion occurs in a minority of PTC patients, ranging from 10% to 20% (10), and it exhibits a mutually exclusive pattern with other driver mutations, including *BRAF* and *RAS* mutations, as well as alternative receptor tyrosine kinase (*RTK*) fusions, indicating *RET* fusion as a pivotal oncogenic event in the pathogenesis of PTC (10).

At present, studies have shown that mutations, including fusions, involving the *BRAF* and *RET* genes are associated with a higher likelihood of a clinical diagnosis of thyroid cancer (11). Simultaneously, several studies have emphasized that the incidence of lymph node and distant metastasis in thyroid cancer exhibiting *BRAF* gene mutations is notably low, suggestive of a potential protective mechanism (12). Conversely, individuals harboring *RET* gene fusion mutations demonstrate a heightened predisposition towards lymph node, nerve, vascular, and distant metastases (13). Additionally, the current investigation of relevant gene mutations of DTC primarily focuses on the prediction of the metastasis to lymph node or bone. As for organ metastasis, particularly specific to the breast, whether it possesses certain characteristics of gene mutations remains uncertain due to the lack of large-scale validation. As a result, a definitive correlation and a theoretical conclusion cannot be drawn at this time. We will try to do some exploration on this aspect. We anticipate that there will be more research in this area in the future.

In accordance with established guidelines (1), all patients diagnosed with DTC ought to undergo neck ultrasonography and serum thyroglobulin (Tg) and thyroglobulin antibody (TgAb) detection within 6 to 18 months after initial therapeutic intervention. For high-risk PTC patients, as exemplified in this case report, if the therapeutic response is favorable, a more rigorous assessment of serum Tg and TgAb levels is paramount to accurately gauge the treatment efficacy pertaining to distant metastases.

We conducted multidisciplinary team consultations with thyroid surgeons and oncologists, and concluded that the patient’s thyroid cancer is currently well-differentiated PTC, and postoperative adjuvant therapy is still based on iodine-131 radiotherapy to treat metastases. However, in view of the large number of metastases and the large range of lesions in patients, we would also recommend that patients undergo external radiotherapy at the relevant site. At the same time, in view of the patient’s pathological type suggesting *BRAF V600E* mutation and *RET* gene mutation, we may suggest that the patient should be treated with vemurafenib, trimetinib, and dabrafenib mesylate to treat *BRAF V600E* mutant tumors. If the above treatment methods fail to achieve satisfactory results, pratinib can be used to treat *RET* fusion thyroid cancer, and sorafenib and other small-molecule multikinase inhibitors targeted the inhibition of Raf kinase.

In summary, despite the generally favorable prognosis of PTC, the potential for distant or even multiple organ metastases underscores the importance of meticulous attention to PTC classification, specific gene expression profiling, postoperative adjuvant therapies (encompassing endocrine therapy, biological therapy, radiotherapy, and others), and rigorous follow-up protocols. Such comprehensive management strategies are vital for ameliorating poor prognostic outcomes associated with this disease.

## Conclusion

Despite thyroid cancer being an endocrine malignancy characterized by high indolence and generally favorable prognosis, it necessitates vigilant attention. In clinical practice, heightened emphasis should be placed on the detection and assessment of multiple genetic markers in patients with PTC. Furthermore, for patients harboring high-risk factors for metastasis, surveillance must encompass not only lymph node and bone metastases but also potential dissemination to other bodily tissues, including the breast. Additionally, we aspire to gain deeper insights into the correlations and underlying mechanisms linking clinical manifestations such as the onset, progression, and metastasis of thyroid cancer with multiple genetic mutations.

## Data Availability

The original contributions presented in the study are included in the article/supplementary material. Further inquiries can be directed to the corresponding authors.

## References

[1] Filetti S Durante C Hartl D Leboulleux S Locati LD Newbold K. Thyroid cancer: ESMO Clinical Practice Guidelines for diagnosis, treatment and follow-up†. Ann Oncol. (2019) 30:1856–83. doi: 10.1093/annonc/mdz400 31549998

[2] Liu Y Wang J Hu X Pan Z Xu T Xu J. Radioiodine therapy in advanced differentiated thyroid cancer: Resistance and overcoming strategy. Drug Resist Updat. (2023) 68:100939. doi: 10.1016/j.drup.2023.100939 36806005

[3] Erdem H Gündogdu C Sipal S. Correlation of E-cadherin, VEGF, COX-2 expression to prognostic parameters in papillary thyroid carcinoma. Exp Mol Pathol. (2011) 90:312–7. doi: 10.1016/j.yexmp.2011.01.008 21335003

[4] Londero SC Krogdahl A Bastholt L Overgaard J Pedersen HB Hahn CH. Papillary thyroid carcinoma in Denmark, 1996-2008: outcome and evaluation of established prognostic scoring systems in a prospective national cohort. Thyroid. (2015) 25:78–84. doi: 10.1089/thy.2014.0294 25368981

[5] Bonichon F Buy X Godbert Y Pointillart V Henriques de Figueiredo B Gangi A. Local treatment of metastases from differentiated thyroid cancer. Ann Endocrinol (Paris). (2015) 76:1S40–6. doi: 10.1016/S0003-4266(16)30013-0 26826482

[6] Elsamna ST Suri P Mir GS Roden DF Paskhover B. The benefit of primary tumor surgical resection in distant metastatic carcinomas of the thyroid. Laryngoscope. (2021) 131:1026–34. doi: 10.1002/lary.29053 32865854

[7] Kharroubi D Richa C Saie C Chami L Lussey-Lepoutre C. Solitary breast metastasis from thyroid papillary carcinoma revealed on whole-body radioactive 131I scan. Clin Nucl Med. (2020) 45:687–8. doi: 10.1097/RLU.0000000000003115 32453076

[8] Zhang D Zhu XL Jiang J. Papillary thyroid carcinoma with breast and bone metastasis. Ear Nose Throat J. (2023) 102:259–62. doi: 10.1177/01455613221145273 36476071

[9] Liu R Xing M. TERT promoter mutations in thyroid cancer. Endocr Relat Cancer. (2016) 23:R143–55. doi: 10.1530/ERC-15-0533 PMC4750651 26733501

[10] Salvatore D Santoro M Schlumberger M. The importance of the RET gene in thyroid cancer and therapeutic implications. Nat Rev Endocrinol. (2021) 17:296–306. doi: 10.1038/s41574-021-00470-9 33603219

[11] Nikiforov YE Nikiforova MN. Molecular genetics and diagnosis of thyroid cancer. Nat Rev Endocrinol. (2011) 7:569–80. doi: 10.1038/nrendo.2011.142 21878896

[12] Xing M. BRAF mutation in papillary thyroid cancer: pathogenic role, molecular bases, and clinical implications. Endocr Rev. (2007) 28:742–62. doi: 10.1210/er.2007-0007 17940185

[13] Wang Z Tang P Hua S Gao J Zhang B Wan H. Genetic and clinicopathologic characteristics of papillary thyroid carcinoma in the Chinese population: high BRAF mutation allele frequency, multiple driver gene mutations, and RET fusion may indicate more advanced TN stage. Onco Targets Ther. (2022) 15:147–57. doi: 10.2147/OTT.S339114 PMC8841610 35173448

